# School Neighbourhood Built Environment Assessment for Adolescents’ Active Transport to School: Modification of an Environmental Audit Tool and Protocol (MAPS Global-SN)

**DOI:** 10.3390/ijerph17072194

**Published:** 2020-03-25

**Authors:** Tessa Pocock, Antoni Moore, Javier Molina-García, Ana Queralt, Sandra Mandic

**Affiliations:** 1 Active Living Laboratory, School of Physical Education, Sport and Exercise Sciences, University of Otago, P.O. Box 56, Dunedin 9054, New Zealand; sandra.mandic@otago.ac.nz; 2 School of Surveying, University of Otago, P.O. Box 56, Dunedin 9054, New Zealand; tony.moore@otago.ac.nz; 3 Department of Teaching of Musical, Visual and Corporal Expression, University of Valencia, Avda. dels Tarongers, 4, 46022 Valencia, Spain; javier.molina@uv.es; 4 AFIPS research group, University of Valencia, 46022 Valencia, Spain; ana.queralt@uv.es; 5 Department of Nursing, University of Valencia, Jaume Roig, s/n, 46010 Valencia, Spain; 6 Centre for Sustainability, University of Otago, P.O. Box 56, Dunedin 9054, New Zealand

**Keywords:** active commuting, educational centre, neighbourhood evaluation, urban environment, walkability, youth

## Abstract

School neighbourhood built environments (SN-BE) can influence adolescents’ active transport to school habits. Typically, SN-BE assessment has involved micro-scale (i.e., environmental audits) or macro-scale (Geographic Information Systems (GIS)) assessment tools. However, existing environmental audits are time/resource-intensive and not specific to school neighbourhoods, while GIS databases are not generally purposed to include micro-scale data. This study evaluated the inter-rater reliability and feasibility of using a modified audit tool and protocol (Microscale Audit of Pedestrian Streetscapes Global–School Neighbourhood (MAPS Global-SN)) to assess the SN-BE of twelve secondary schools in Dunedin, New Zealand. Correlations between MAPS Global-SN and GIS measures of the SN-BE were also examined. Specifically, MAPS Global-SN audit and GIS spatial analysis (intersection density, residential density, land use mix, walkability) was conducted within a 0.5 km street-network buffer-zone around all twelve schools. Based on investigator and expert consultation, MAPS Global-SN included eight modifications to both auditing processes and items. Inter-rater reliability data was collected from two independent auditors across two schools. The feasibility of a condensed audit protocol (auditing one side of each street segment in the neighbourhood, compared to both sides) was also assessed. Results indicated the modified MAPS Global-SN tool had good to excellent inter-rater reliability and the condensed MAPS Global-SN audit protocol appeared to sufficiently represent the micro-scale SN-BE. Results also highlighted the complementary nature of micro- and macro-scale assessments. Further recommendations for SN-BE assessment are discussed.

## 1. Introduction

A physically active lifestyle is associated with well-known health benefits [1]. Yet, many adolescents are not sufficiently active to meet current physical activity guidelines of 60 min daily moderate-vigorous physical activity [2]. Walking or cycling to (and from) school (active transport to school; ATS) has the potential to contribute towards adolescents’ daily moderate-vigorous physical activity [3], particularly for adolescent girls [4]. Despite possible physical activity contributions, many countries report decreased rates of adolescents’ ATS over recent decades [5,6,7,8]; although such reports vary considerably between countries [9].

Supportive home and school neighbourhood built environments (SN-BE), characterised by the integration of transportation systems, diverse land use distributions, designs and functions [10,11], afford access and opportunity for ATS [12]. Built environment characteristics are generally classified as either micro-scale (e.g., pedestrian/cycling infrastructure, intersection design, street amenities) or macro-scale (e.g., street connectivity, residential density, land use mix) attributes [13,14,15]. Both micro-scale and macro-scale attributes of the home and SN-BE influence adolescents’ ATS behaviours and perceptions of safety [16,17]. Furthermore, parental concern over micro-scale built environment attributes, namely traffic, crime and personal safety, may also shape adolescents’ transport mode choice [16,18,19,20].

Considerable research among children and adolescents reflects home neighbourhood built environment influences on ATS, indicating higher odds of ATS when children and adolescents perceive they live in aesthetically-pleasing [21,22] and safe neighbourhoods, with slow traffic speed (especially among adolescent girls) [23], and diverse and good public services [21,23] (micro-scale attributes). In addition, odds of ATS are greater when adolescents live in high-income and high-walkability neighbourhoods [19], with higher intersection density [24,25], and shorter distances to school [22,24,25,26] (macro-scale attributes). However, limited research and assessment of the SN-BE has been conducted in relation to ATS among adolescents e.g., [17,27]. When considering the SN-BE, prevalence and frequency of ATS among rural adolescents is reportedly associated with micro-scale attributes of the SN-BE, including sidewalk continuity, presence of curbs, and the height, continuity and setbacks of buildings [27]. In addition, density of major roads and public transportation facilities along the route to school [28] and concern over safety of walking to school [17] have been associated with reduced odds of adolescents cycling to school or using ATS, respectively.

Macro-scale attributes of the neighbourhood built environment, such as measures of density (e.g., residential or intersection densities) and composite measures of environmental supportiveness for physical activity (e.g., walkability index), are frequently computed through Geographic Information Systems (GIS) analysis when ATS is analysed [24,26,29,30]. In contrast, micro-scale environmental data are usually quantified through observational environmental audit tools which assess attributes relevant to walking and cycling [31]. Environmental audit tools, for example, MAPS Global [32], PEDS [33] and SPACES [34], have typically been conducted in-person by physically navigating neighbourhood streets. However, considerable content heterogeneity exists between audit tools [31], as well as in protocols defining street sampling methods and neighbourhood boundaries for assessing the SN-BE e.g., [35,36,37].

The Microscale Audit of Pedestrian Streetscapes (MAPS) Global audit tool is among the latest developments in micro-scale built environment assessment and has been designed for international use, based on earlier tools [32]. This instrument reports generally high inter-rater reliability in different countries [32,38]. MAPS Global has been designed to measure detailed streetscape features relevant to physical activity, drawing on concepts from previously developed questionnaires and audit tools to assess micro- and macro-scale environmental attributes along a participant’s route (from home to destination). MAPS Global audit protocols have also been developed to enable micro-scale assessment of an entire neighbourhood [32], mainly the participant’s home neighbourhood environment. However, as a proposed alternative to the time- and resource-intensive in-person audits often required for a neighbourhood, further research suggests sampling 25% of neighbourhood street segments (the road between two intersections) (i.e., within a 400 m radius) may be sufficient to represent the pedestrian environment [39]. 

The present study had three aims: (1) to present a modified MAPS Global audit tool and protocol, specific for SN-BE assessment (rather than for assessing a participant’s route), and to evaluate the inter-rater reliability of the modified tool; (2) to assess whether a condensed audit protocol using the modified MAPS Global tool (auditing one side of each street segment in the school neighbourhood, compared to both sides) sufficiently represented the micro-scale SN-BE of twelve secondary schools in Dunedin, New Zealand; and (3) to explore correlations between the modified MAPS Global tool and GIS measures of the SN-BE, since previous studies have shown that both micro- and macro-scale attributes of the built environment are associated with ATS [16,23,24]. This study also provides recommendations for future SN-BE assessment using the modified MAPS Global tool.

## 2. Materials and Methods

### 2.1. Study Context

The present study extends the cross-sectional Built Environment and Active Transport to School (BEATS) Study (2014–2017) [30], conducted in the coastal city of Dunedin, New Zealand (population ~120,000). Through qualitative (focus groups, interviews) and quantitative (surveys, GIS analysis, accelerometery, route to school mapping) methods, the BEATS Study examined determinants of Dunedin adolescents’ ATS from perspectives of students, parents, teachers and principals across all twelve Dunedin secondary schools [30,40]. Dunedin secondary schools varied in size (range: 360–872 students), school decile (measure of students’ socio-economic status; 1 = most deprived to 10 = least deprived) (range: decile 5–10), co-educational (n = 5) or single-sex status (n = 7), and topography (hilly = 5 schools; flat/gentle slope = 7 schools). Based on BEATS Study data, the optimal threshold distance for walking to school among Dunedin adolescents is ≤2.25 km [17]. Rates of ATS among Dunedin adolescents living ≤2.25 km from school ranged from 47.8% to 70.0% per school (average: 60.7%) [17].

### 2.2. Procedures

Micro- and macro-scale SN-BE attributes were assessed using the MAPS Global tool [32] and GIS-based spatial analysis, respectively. The following sections are arranged in line with the three study aims.

### 2.3. School Neighbourhood Built Environment Audit Using MAPS Global Tool

**Figure 1:**
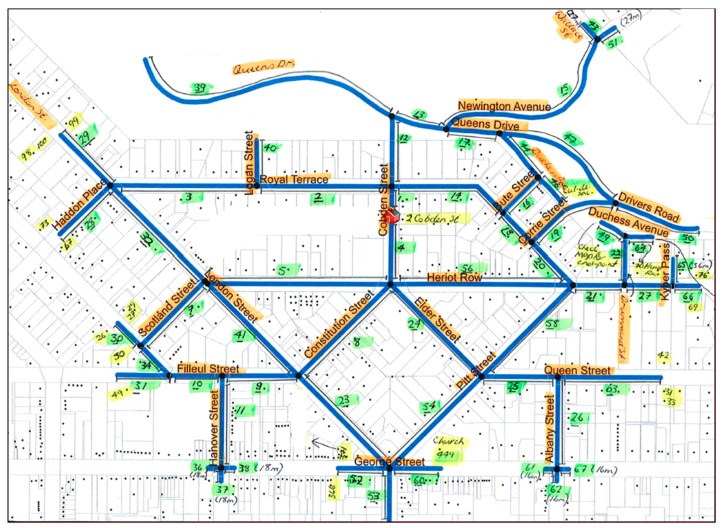
Example worksheet of the 0.5 km street network buffer-zone (blue lines) used for conducting route and segment MAPS Global audits. Note. Green highlighting refers to the identifying code of the segment; yellow highlighting refers to a street address point; black dots refer to residences.

MAPS Global environmental audit (Version 3/2/2016; available from https://drjimsallis.org/measure_maps.html) [32] was physically conducted within a 0.5 km street-network buffer-zone around each Dunedin secondary school, as described previously [17]. A 0.5 km street-network buffer-zone was selected in line with previously published protocols for home neighbourhood built environment assessment in children and adolescents [41,42] and due to the time-intensive nature of environmental audits. Geocoded school address points served as the origin of a 0.5 km street-network service area (or network buffer) in Esri ArcGIS 10.4 (Environmental Systems Research Institute, Redlands, CA, USA). Dunedin city streets were represented by the road dataset obtained from the New Zealand national mapping agency, Land Information New Zealand (LINZ, Wellington, New Zealand), with the required network connectivity subsequently calculated on that dataset. The street-network buffer-zone was calculated along the road network and included publicly accessible roads and one-way streets. Individual street segments (road between two intersections) were obtained by splitting lines by junction points from the road network dataset in ArcGIS. Intersections within the street-network buffer-zone with ≥ 3 connecting roads were selected (to filter out any ‘false’ intersections of two connecting roads that may be present in the dataset). Identified school neighbourhood street segments, intersections and connecting roads were assigned a unique identifier code. Identifying information for each street segment was then duplicated to reflect odd and even street sides based on address points. Identifier codes allowed easy differentiation of street segments within each school neighbourhood (Figure 1) and assisted with subsequent integration of MAPS Global data into a geographic information database.

Prior to commencing MAPS Global auditing, all generated street segments and intersections were checked and corrected as necessary. To achieve a more comprehensive and reflective assessment of micro- and macro-scale SN-BE attributes, the following decisions related to generated street-network buffer-zones were made:

(1) To audit a street segment at the boundary of the 0.5 km buffer-zone, at least one residence must have been present. Identifier codes of segments containing no residences (range: 1–21 m in length) were noted and excluded from the audit. Sixty individual street segments (6.0% of total street segment sample) were excluded based on this criterion.
(2) Partial buffer coverage from both ends of the street segment was extended (i.e., to create one full segment) when at least one residence was present at each end and the majority of the street segment length was included in the original buffer. Extension of the street segment length to create one full segment was completed four times for buffer coverage breaks 20–90 m in length.

**Figure 2:**
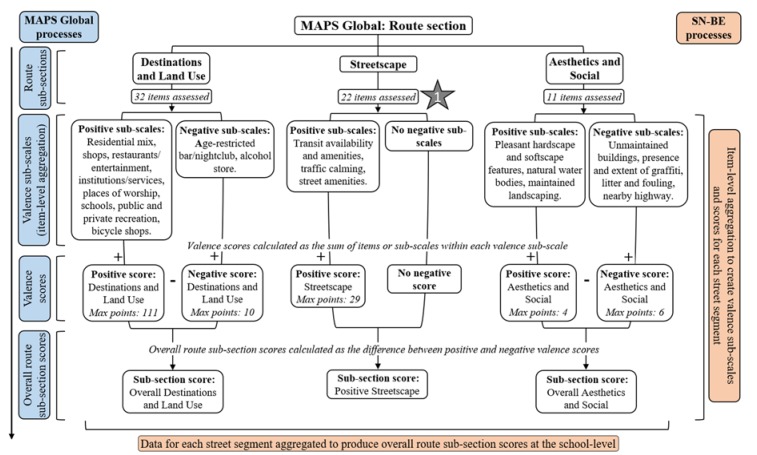
Hierarchical scoring system of MAPS Global route section and sub-sections (adapted from [43]). Note. Star denotes number of item-level modifications. Max points refers to the maximum number of points available from the summation of sub-scales.

**Figure 3:**
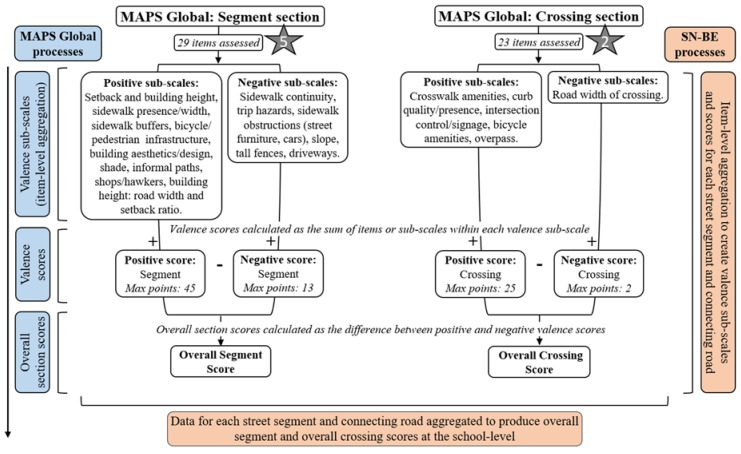
Hierarchical scoring system of MAPS Global segment and crossing sections (adapted from [43]). Note. Star denotes number of item-level modifications. Max points refers to the maximum number of points available from the summation of sub-scales.

The MAPS Global audit tool was used to assess routes (sub-sections: destinations and land use, streetscape, and aesthetics and social), segments, crossings and cul-de-sacs [32] within each school neighbourhood. Briefly, route audits assessed destinations, street amenities and built environment characteristics (Figure 2), while segment audits assessed micro-scale elements of the street layout, sidewalks, buildings and bicycle facilities (e.g., the presence and quality of bicycle lanes/zones) (Figure 3). The crossing audit assessed aspects of intersection control, traffic light signalisation, pedestrian protection and crosswalk treatment (Figure 3), and, when present, cul-de-sac audits assessed amenities available in cul-de-sacs.

#### 2.3.1. MAPS Global Audit and Protocol Modifications

Modification of several MAPS Global auditing processes, items and data coding were necessary to enable this tool to be used for assessment of the school neighbourhood, rather than assessment of a participant’s route. Discussion and agreement between study investigators (T.P., A.M., S.M.) and expert consultation (with A.Q.; a part of the team who developed the original MAPS Global tool) was completed prior to implementing changes. At the process-level, route and segment audits were completed along both sides of each pre-determined street segment (labelled odd and even sides), crossing audits were conducted across all connecting roads in an intersection of ≥3 connecting roads and cul-de-sac audits only assessed available amenities (e.g., basketball hoops) (Table 1). In addition, the following processes were observed when completing the crossing audit: (1) driveways present at an intersection were not counted as pre-/post-crossing curb ramps; (2) the street side audited as the pre-crossing curb was noted on a school neighbourhood map to allow input into a geographic information database; (3) all street segments were audited in their entirety regardless of footpath presence/absence (i.e., unanticipated mid-segment crossing audits were not applicable for the SN-BE); (4) all crossings were audited regardless of a footpath on the opposite street side; and (5) audits of crossings that did not have pedestrian infrastructure in place (considered as ‘unofficial’ crossing points) were arbitrarily assessed within 5 m of the street corner, regardless of whether a barrier prevented the crossing from happening here (i.e., flower bed, parked cars). At the item-level, one modification was necessary in the streetscape sub-section of the route audit to reflect transport facilities available to adolescents. In addition to counting public transit stops (as used in the original tool), school bus stops were also recorded if they were present and close to a school entrance. Five segment items (residential or commercial street, building height, high and low streetlights) were modified to assess both street sides as discrete segments, rather than as a combined unit as intended with the original tool. The crossing audit involved modification of two items: assessment of intersection control at each connecting road in an intersection and the addition of fluorescent orange disks/round flashing orange lights at pedestrian crossings as potential crossing aids (applicable to a New Zealand context) (Figure 2 and Figure 3). Minor modifications applied to MAPS Global data coding are available in online Supplementary Material.

#### 2.3.2. Auditing Processes

MAPS Global audits of all twelve Dunedin secondary schools were completed between September 2017 and January 2018 by T.P. (Table 1). A sample of segments and crossings within neighbourhoods of the first two schools were independently assessed by two auditors (route (n = 44), segment (n = 44) and crossing (n = 92)), at the same time and on the same day, and make up the data for inter-rater reliability assessment. These first two schools were selected based on proximity to the auditors’ location. Results were compared to ensure consistency and inter-rater reliability assessment was completed (see results section for details). Discrepancies were discussed and consensus reached by an in-person re-audit of those school neighbourhoods. Co-audits were discontinued due to time and resource constraints.

School audits were completed by auditing crossings first (1–3 days per school), followed by route, segment and cul-de-sac audits over a 1–3-week period per school. Audit time was dependent on weather and the number of intersections and street segments present in the school neighbourhood. Route, crossing and cul-de-sac data were recorded on paper audit forms (available from https://drjimsallis.org/measure_maps.html). Segment data was directly entered into a pre-designed MAPS Global database in Microsoft Excel (Microsoft, Redmond, WA, USA) using an iPad Mini (Apple Inc., Cupertino, CA, USA). Overlapping area between the schools was manually identified, audited once and copied to relevant schools to create a full MAPS Global dataset for each school. Paper audits were entered into Microsoft Excel databases and double-checked by T.P. for accuracy. iPad-based audit entries were checked by T.P. to ensure all item data was filled in and consistent with MAPS Global data coding (Version September 6, 2017) [44].

On average, audited street segments were 114.1 ± 73.6 m in length (range per school: 11.5–556.6 m; maximum length >0.5 km resulted from the merging of segments either side of school address point) and totalled 106.6 km when each side of all street segments were summed together (average per school: 8.9 km; range per school: 2.7–14.0 km) [17]. Crossing audits included 516 (67.3%) audits at three-way intersections, 224 audits (29.2%) at four-way intersections, 15 audits (2.0%) at five-way intersections, and 12 audits (1.6%) at six-way intersections [17].

#### 2.3.3. MAPS Global Scoring System

Calculation of MAPS Global sub-scales and scores are described in detail elsewhere [32,44]. Briefly, and in relation to auditing the SN-BE, Figure 2 and Figure 3 depict the hierarchical scoring system of MAPS Global (calculated for each street segment and connecting road within a school neighbourhood), in which route, segment and crossing audit items are summed into sub-scales and sub-scales into scores. Cul-de-sac sub-scales did not contribute to MAPS Global scoring. Average school-level data was produced by aggregating overall section scores for all route, segment and crossing audits within each school neighbourhood. Further aggregation of school-level item and sub-scale data produced overall grand scores and cross-domain sub-scales for each school.

Overall grand scores for each school were produced from the sum of valence (positive and negative) sub-scale scores from the main MAPS Global sections (route (destinations and land use, streetscape, aesthetics and social), segment, crossing), developed based on the anticipated sub-scale effect on physical activity (maximum points: 210). Cross-domain sub-scales of pedestrian infrastructure (e.g., items: sidewalk continuity, marked crosswalks), pedestrian design (e.g., building setbacks, pedestrian countdown signals, crossing aids) and bicycle facilities (e.g., bicycle lane presence/quality) were calculated from the summation of school-level averages of route, segment and crossing items. Each cross-domain sub-scale was scored out of a maximum number of points based on the sum of individual items (maximum points: pedestrian infrastructure = 27; pedestrian design = 22; bicycle infrastructure = 11). A higher score indicated a more supportive environment for walking or cycling. MAPS Global sub-scales and overall scores were computed using SPSS version 24.0 (IBM Corp., Armonk, NY, USA).

### 2.4. Condensed MAPS Global Audit Protocol

The modified MAPS Global audit tool was also used to assess whether a condensed audit protocol (auditing one side of each street segment within each school neighbourhood, compared to both sides) sufficiently represented the micro-scale SN-BE of the twelve secondary schools in Dunedin, New Zealand. In preparation for examining correlations between odd and even sides of each street segment, MAPS Global route, segment, overall grand and cross-domain sub-scale scores were calculated individually for odd and even street sides. However, as all connecting roads to an intersection were assessed, it was not possible to distinguish crossing audits as originating from odd and even street sides. Crossing scores were instead calculated as the sum of all assessed crossings within each intersection of each school neighbourhood. The overall grand score and cross-domain sub-scales for odd and even sides of each street segment were computed using the same values for relevant crossing data.

### 2.5. Macro-Scale GIS Analysis of the School Neighbourhood Built Environment

Macro-scale GIS analytical procedures for the present study have been described in detail elsewhere [17]. Briefly, intersection density (junctions/km^2^), residential density (residences/km^2^), land use mix (ranges between 0 and 1; homogeneity and heterogeneity in land uses, respectively) and a composite walkability index (calculated as the z-scores of intersection density, residential density and land use mix) [46] were computed for each SN-BE. GIS-derived variables were applied to a 0.5 km street-network buffer-zone around each school (detailed polygon-generated service areas, no trim) and corresponded to the street-network buffer-zone distance used in MAPS Global audits. Residential density calculations assumed a single residence was occupied at a single address point. Land use mix was computed using line-generated service areas aggregated from 0.095 km buffers from the road centreline and used Dunedin City Council land use reference data (including residential, commercial, industrial, tertiary campus and open space). GIS measurements were derived from Dunedin City Council and Land Information New Zealand (LINZ) spatial data.

On average, the twelve SN-BEs varied in intersection density (range: 6.2–100.8 junctions/km^2^; mean: 53.8 ± 26.0 junctions/km^2^) and residential density (range: 62.2–1,334.5 residences/km^2^; mean: 908.7 ± 343.5 residences/km^2^) and had low land use heterogeneity (range: 0.0–0.6; mean: 0.3 ± 0.2) [17]. The composite walkability index ranged across schools from −2.6 to 4.0 (mean: 0.0 ± 1.7) [17].

### 2.6. Data Analysis

To assess inter-rater reliability, the intraclass correlation coefficient (ICC) was calculated for the computed MAPS Global sub-scales and scores. The ICC was classified to indicate test-retest reliability that was: “excellent” (ICC ≥ 0.75), “good” (0.60–0.74), “fair” (0.40–0.59) and “poor” (<0.40) [47]. MAPS Global data and GIS-derived variables were analysed using Pearson’s Product Moment Correlations to examine correlations between, (1) odd and even sides of each audited street segment and (2) between MAPS Global sub-scales and scores and GIS-measured SN-BE attributes. Two MAPS Global variables (route sub-section: destinations and land use; cross-domain sub-scale: bicycle facilities) were not normally distributed and were analysed using Spearman’s Rank Correlation Coefficient. Data were analysed using SPSS version 24.0 (IBM Corp., Armonk, NY, USA).

## 3. Results

As shown in Table 2, evaluation of inter-rater reliability of this MAPS Global tool modification indicated all sub-scales and overall scores had "good" or "excellent" reliability (ICCs = 0.60 to 0.99), considering the Cicchetti criteria [47]. The aesthetics and social sub-section showed lower ICC than other sub-scales, but reliability was still in the “good” range (ICC ≥ 0.60).

Table 3 presents descriptive data on MAPS Global measures of the SN-BE within a 0.5 km street-network buffer-zone. Overall, MAPS Global overall grand score and cross-domain sub-scales (pedestrian infrastructure, pedestrian design, bicycle facilities) showed limited variation across the twelve schools. Significant positive correlations between identified odd and even street segment sides were observed for the MAPS Global overall grand score, pedestrian design sub-scale, route section sub-scales (positive streetscape, aesthetics and social) and the overall segment score (r = 0.68 to r = 0.98; all *p* < 0.05). No significant correlations were found between identified odd and even street segment sides for the pedestrian infrastructure, bicycle facilities or destinations and land use sub-scales. 

Examination of the association between MAPS Global and GIS measures of the SN-BE showed that only the cross-domain pedestrian design sub-scale from the MAPS Global tool was positively correlated with school-level GIS measures of intersection density (r = 0.66, p = 0.020), residential density (r = 0.64, *p* = 0.025) and overall walkability (r = 0.75, *p* = 0.005) (data not tabulated).

## 4. Discussion

The present study described modifications to the MAPS Global audit tool required for school neighbourhood assessment and assessed the inter-rater reliability of these modifications, examined whether auditing one side of each street segment provided a sufficient representation of the micro-scale SN-BE of Dunedin, New Zealand secondary schools, and explored correlations between MAPS Global and GIS measures of the SN-BE. Overall, results indicate that (1) MAPS Global tool modification showed "good" to "excellent" inter-rater reliability of sub-scales and overall scores, (2) auditing one side of the street segment using the modified MAPS Global tool and condensed audit protocol could be a feasible alternative to represent the micro-scale SN-BE, and (3) SN-BE assessment using MAPS Global tool should be complemented with GIS measures of the SN-BE. Given the present findings, we recommend the use of the modified MAPS Global tool and condensed audit protocol (henceforth, MAPS Global—School Neighbourhood (MAPS Global-SN)) for simplifying assessment of school neighbourhoods in future studies. Further recommendations for SN-BE assessment are discussed below.

Building on the MAPS Global audit tool, the latest international development in micro-scale built environment assessment, MAPS Global-SN audit tool and protocol modifications have broadened the original scope of assessment intended with MAPS Global. In particular, MAPS Global-SN focused on the larger-scale school neighbourhood within a 0.5 km street-network buffer-zone, rather than on a participant’s route or home neighbourhood environment [32]. In addition, item modification enabled the assessment of additional items relevant to the SN-BE; specifically, the inclusion of school bus stops as public transit stops and fluorescent orange disks at pedestrian crossings as crossing aids (the latter being applicable to a New Zealand context). The modified MAPS Global-SN tool showed good to excellent evidence of inter-rater reliability for assessed sub-scales and overall scores, consistent with reports of generally high inter-rater reliability in different countries [32,38]. The lowest reliability was found for the aesthetics and social characteristics sub-section of the route audit (ICC = 0.60), although reliability was still in the "good" category. The lower ICC value for the aesthetics and social section compared to other sections is consistent with previous research [32,38]. As previously indicated [32], items from MAPS Global that are related, for example, with landscaping, water features or dog excrement (as included in the aesthetics and social sub-section), require more subjective judgment than other kinds of items. Nonetheless, inter-rater reliability assessment suggests that results from MAPS Global-SN are also reliable when assessed by different individuals in a New Zealand context.

The condensed MAPS Global-SN audit protocol (auditing one side of each street segment) appeared to sufficiently represent the micro-scale SN-BE of twelve Dunedin secondary schools, indicating MAPS Global-SN tool and condensed protocol may be a feasible alternative to micro-scale SN-BE assessment. Significant positive correlations between odd and even street sides were observed for the overall grand score, pedestrian design sub-scale, route section sub-scales (positive streetscape, aesthetics and social) and overall segment score. Positive correlations indicate the presence of similar environmental features on both sides of each street segment in the urban city environment this study was conducted in. Such findings have important implications for simplifying future school neighbourhood audits and potentially reducing the time spent auditing schools by half (when compared to auditing both sides of each street segment, as in the present study). Furthermore, these findings contribute to discussions on appropriate street sampling protocols to represent the built environment e.g., [31,36,37,39].

No significant correlation was found between odd and even street sides for the bicycle facilities sub-scale, likely due to the low proportion of schools with bicycle infrastructure in their neighbourhoods and presence of built cycle paths on only one side of the road (going in only one direction) near some schools. In addition, no significant correlations were found between odd and even street sides for pedestrian infrastructure and destinations and land use sub-scales in the present study. These findings could be explained by the mixture of residential and commercial land uses within the urban city environment of Dunedin. However, as pedestrian infrastructure and destinations and land use amenities likely affect ATS among adolescents i.e., [16,19,23], future studies may need to consider the local urban environment around schools when deciding whether to complete their SN-BE assessments on one side of the street, keeping in mind the present findings which indicate such assessment may not produce the same results for pedestrian infrastructure, bicycle facilities and destinations and land use sub-scales as assessment completed on both sides of the street.

Results of the present study suggest that MAPS Global-SN and GIS measures of the SN-BE evaluate built environment attributes at different scales (i.e., micro-scale and macro-scale levels), indicated by few significant associations between the two assessment tools. In relation to ATS, extensively studied macro-scale GIS measures of the home neighbourhood built environment indicate higher intersection density [24], high-income and high-walkability neighbourhoods [19] and shorter distances to school [22,24,26] are associated with greater odds of ATS among adolescents. The development of various micro-scale environmental audit tools, including the original MAPS Global, reflects the awareness that smaller environmental details are also likely to influence physical activity experiences and behaviours in an environment [31], including ATS behaviours [27,28,48]. Currently, environmental audits collect data on predominantly micro-scale environmental attributes that are typically not captured in GIS databases [31,49]. For example, GIS databases are not generally purposed to include micro-scale data related to environmental aesthetics or social function/dysfunction. Environmental aesthetics and social characteristics may be more difficult to reliably audit (as demonstrated in the present study with the aesthetics and social sub-section showing only “good” inter-rater reliability) and quantify; however, such characteristics are related to ATS behaviours in adolescents [19,21]. Therefore, results of the present study highlight the potential complementary nature and added value of combining micro- and macro-scale SN-BE assessment to understand the role of the school neighbourhood in encouraging physical activity in adolescents, particularly through supporting ATS.

### 4.1. Recommendations and Considerations

Reflections on the modified MAPS Global-SN tool, condensed protocol and data collection processes have resulted in the formulation of three recommendations and four considerations for future studies assessing the SN-BE.

#### 4.1.1. Recommendations Regarding the Modified MAPS Global-SN Tool, Condensed Protocol and Data Collection Processes

(1) Audit one side of each street segment when using the modified MAPS Global-SN tool and condensed protocol. Based on results of the present study, this recommendation is applicable to the New Zealand context. Future studies should also assess the appropriateness of using the modified MAPS Global-SN tool and condensed protocol in different geographical contexts, while considering the unique micro-scale environmental attributes.
(2) Use a combination of micro- and macro-scale built environment assessment tools, which are sensitive to the scale of items being assessed. For example, micro-scale assessment tools, such as MAPS Global-SN, should include information on environmental aesthetics and social function; while macro-scale assessment tools, such as GIS analysis, should include information on density and composite measures of environmental supportiveness for physical activity (i.e., walkability index).
(3) Set a maximum segment length limit, after which a new route and segment audit is started. In the present study, potential maximum segment length was 0.5 km when following the street network. However, it was often difficult to keep track of the number of items for aggregation (i.e., number of street trees present) or compare attributes (i.e., proportion of properties protected by gates/walls/tall fences) over a long segment length. Drawing from the study of Clifton, Smith and Rodriguez [33], a suggested alternative to the 0.5 km maximum segment length is to subdivide segments if they are longer than 0.2 km (the original value reported was 700 ft (~0.21 km)) to maintain a consistent segment length and enable accurate comparisons of the variation in micro-scale attributes across segments. For example, based on the 0.2 km length recommendation, we suggest the following equal and objective subdivisions: divide the segment in half if it is between 0.2 km and 0.4 km in length and in thirds if the segment is between 0.41 km and 0.6 km in length.

#### 4.1.2. Considerations for Future Studies

(1) Researchers using MAPS Global-SN or MAPS Global to audit a neighbourhood environment should consider an audit protocol that is proportional to the time and resources available to the research team e.g., [35,36,39].
(2) Researchers should consider using measures of traffic speed and traffic volume as a part of school neighbourhood assessment. The route section of MAPS Global-SN assesses traffic calming characteristics (e.g., signs, speed humps), while the segment section assesses the number of traffic lanes as a proxy for traffic volume. However, as traffic safety is a key concern for adolescents and their parents [23,50], particularly for cycling to school [16,51], a more accurate measure of traffic speed on street segments and traffic volume in the school neighbourhoods may be beneficial in understanding ATS behaviours. Such measurements should be performed at a specific time of the day with respect to the research question. For example, studies examining ATS in children and adolescents should consider assessing traffic speed and volume in the school neighbourhood during peak times for school commuting.
(3) Future studies should consider using MAPS Global-SN to assess the SN-BE across a range of geographic settings and should also consider including items which are applicable to the local context being studied.
(4) The modified MAPS Global-SN tool and protocol has been used to explore associations between the micro-scale SN-BE and ATS in adolescents, in a New Zealand context [17]. Researchers should continue this work across a range of geographic settings to establish a connection between the micro-scale and macro-scale SN-BE and ATS in adolescents, extending the vast literature available in children e.g., [35,36,37]. In addition, future research could explore associations between MAPS Global-SN and adolescents’ physical activity behaviours during the trip to/from school, using methods such as questionnaires and/or accelerometry.

### 4.2. Strengths and Limitations 

Study strengths include a novel approach to SN-BE assessment, building on the latest developments in micro-scale built environment assessment designed for international use (MAPS Global tool), complementing micro-scale assessment with macro-scale GIS assessment of the SN-BE, reliability testing of the modified tool, and generating recommendations and considerations for future studies assessing the neighbourhood using MAPS Global-SN tool and condensed protocol. Study limitations include data collection in one city, school neighbourhood assessment over a 0.5 km street-network buffer-zone (due to time- and resource-constraints), assessing only one type of condensed audit protocol for micro-scale SN-BE assessment, and using the same crossing score value when examining correlations between each side of the street segment for the overall grand score and cross-domain sub-scales (as it was not possible to distinguish crossing audits as originating from odd and even street sides). In addition, device-measured physical activity specific to transport to/from school in adolescents was not assessed in this study. Finally, school neighbourhood environmental scans were completed using only the modified MAPS Global-SN audit tool and protocol. Therefore, we could not compare physical activity/ATS associations between the original MAPS Global tool and the modified MAPS Global-SN audit tool and protocol.

## 5. Conclusions

The relationship between the SN-BE and ATS in adolescents is an area in need of further development. The modified MAPS Global-SN tool and condensed protocol presents a potentially feasible alternative to micro-scale SN-BE assessment, simplifying data collection procedures, and reducing the time and resource commitment required to audit the school neighbourhood. As attributes of the built environment, at the micro- and macro-scale level, influence ATS behaviours, it appears important that complementary micro-scale environmental audits and macro-scale GIS measures are both used in SN-BE assessment. Future studies should consider SN-BE audit protocols that include locally relevant assessment items and are proportional to the time and resources available. In addition, future studies should elaborate the relationship between the micro-scale and macro-scale SN-BE and ATS in adolescents.

## Figures and Tables

**Table 1:** Comparison between original MAPS Global auditing processes and modification of MAPS Global for assessment of school neighbourhood.

MAPS Global Section	Original MAPS Global Audit Tool	Modified MAPS Global Audit Tool	Total Number Assessed	Range per School
Route	***Assesses: Destinations, amenities and characteristics of the walked route***		934	10–160
	Origin of route audit: Participant’s home address. Audited length: 0.25–0.45 miles (~0.40–0.72 km) from participant’s home towards nearest pre-determined commercial destination. Route assessment stops at the intersection following 0.25-mile threshold or mid-segment at 0.45-miles in the absence of an intersection. Route section completed once per participant’s route to assess the entire walked route. Overall summary provided for entire walked route by considering both sides of all street segments together.	Origin of route audit: School address point. Audited area: 0.5 km street-network buffer-zone branching out from school address point. Route assessment stops immediately where the 0.5 km street-network buffer-zone ends (exception: partial buffer coverage or absence of a residence, as stated above). * Route section completed separately for odd and even street segment sides within the buffer-zone. Multiple route section audits per school. Individual summary provided for each side of each street segment walked.		
Segment	***Assesses: Micro-scale features of street layout, sidewalks, buildings and bicycle facilities***		934	10–160
	Each participant’s route made up of one or more segments (dependent on road layout around each home address). Single side of the street along a participant’s route is assessed.	Each school neighbourhood made up of many segments (dependent on road layout around each school). Both sides of each street segment are assessed individually.		
Crossing	***Assesses: Intersection control, traffic light signalisation, pedestrian protection and crosswalk treatment***		767	3–118
	Located between two street segments. Crossings assessed when the auditor passes through an intersection along the pre-determined route. Some pre-determined routes may not have crossings.	Located between two street segments. All intersections (≥3 connecting roads) within the school neighbourhood are assessed. Within each intersection, all connecting roads are assessed. Multiple crossing section audits completed per school.		
Cul-de-sac	***Assesses: proximity to participant’s home, physical activity/recreation amenities and surveillance***		14	0–6
	Cul-de-sac must be within 400 ft (120 m) of participant’s home to be assessed.	All cul-de-sacs within the school neighbourhood assessed. Only amenities question assessed. Proximity and surveillance not applicable to assessment of the school neighbourhood.		

Note. Original MAPS Global auditing processes are found in Geremia and Cain [45]. * A maximum segment length was not considered necessary when developing the modified audit protocol, because the MAPS Global tool did not include a maximum segment length (other than the 0.45-mile threshold). However, auditing a 0.5 km segment meant it was often difficult to compare different characteristics across the entire segment and to keep track of the total number of items present (i.e., number of street trees). As a recommendation of this study, a shorter maximum segment length is proposed in the discussion section.

**Table 2:** Inter-rater reliability of MAPS Global sub-scales and scores.

	ICC	CI 95%	Rater’s Mean ± SD	
			Rater 1	Rater 2
Overall Grand score	0.97	−15.22, 1.00	17.48 ± 2.80	18.63 ± 3.85
Cross-domain sub-scales				
Pedestrian Infrastructure	0.80	−131.53, 1.00	6.28 ± 0.10	6.07 ± 0.26
Pedestrian Design	0.98	−12.14, 1.00	3.96 ± 2.61	4.50 ± 3.47
Bicycle Facilities	0.99	0.29, 1.00	1.17 ± 1.37	1.10 ± 1.47
Route section sub-scales				
Destinations and Land Use	0.93	0.86, 0.96	2.45 ± 2.28	2.45 ± 2.57
Positive Streetscape	0.93	0.88, 0.96	2.05 ± 2.45	1.98 ± 2.31
Aesthetics and Social	0.60	0.26, 0.78	−0.61 ± 1.57	0.09 ± 1.27
Segment section sub-scales				
Overall segment score	0.73	0.50, 0.85	11.80 ± 4.36	12.61 ± 4.73
Crossing section sub-scales				
Overall crossing score	0.99	0.99, 1.00	2.33 ± 3.26	2.29 ± 3.18

Note. ICC = Intraclass Correlation Coefficient; CI = Confidence Interval; SD = Standard Deviation.

**Table 3:** MAPS Global sub-scales and scores across odd and even sides of street segments, presented individually and combined.

	MAPS Global Scores						
	ODD Street Side		EVEN Street Side			COMBINED	
	Mean ± SD	min, max	Mean ± SD	min, max	r-Value (*p*-Value)	Mean ± SD	min, max
Overall grand score	15.69 ± 1.96	12.62, 19.23	15.71 ± 2.48	11.13, 19.80	0.89 (<0.001) **	15.70 ± 2.16	12.23, 19.52
Cross-domain sub-scales							
Pedestrian infrastructure	6.70 ± 0.67	5.57, 7.96	6.39 ± 0.88	4.60, 7.57	0.55 (0.067)	6.54 ± 0.68	5.60, 7.76
Pedestrian design	3.11 ± 1.10	1.46, 4.93	3.17 ± 1.27	1.26, 5.21	0.98 (<0.001) **	3.14 ± 1.18	1.36, 5.07
Bicycle facilities	0.43 ± 0.79	0.00, 2.08	0.28 ± 0.68	0.00, 2.20	0.43 (0.160)	0.44 ± 0.81	0.00, 2.04
Route section sub-scales							
Destinations and land use	1.78 ± 0.49	0.00, 17.00	1.78 ± 0.73	0.00, 16.00	0.51 (0.090)	1.78 ± 0.59	0.00, 17.00
Positive streetscape	1.29 ± 0.55	0.00, 13.00	1.58 ± 0.80	0.00, 12.00	0.68 (0.015) *	1.43 ± 0.62	0.00, 13.00
Aesthetics and social	−0.50 ± 0.44	−4.00, 3.00	−0.46 ± 0.66	−4.00, 3.00	0.71 (0.009) **	−0.48 ± 0.51	−4.00, 3.00
Segment section sub-scales							
Overall segment score	10.60 ± 1.22	−4.00, 22.00	10.29 ± 2.05	−2.00, 23.00	0.83 (0.001) **	10.45 ± 1.56	−4.00, 23.00
Crossing section sub-scales							
Overall crossing score	-	-	-	-	-	2.52 ± 1.09	−2.00, 15.00

Note. Statistically significant differences are bolded. * *p* < 0.05, ** *p* < 0.01. SD = Standard Deviation. Mean, minimum (min) and maximum (max) for each initial sub-scale (route, segment, crossing) comes from individual school values. Mean, min and max from the overall grand score and cross-domain sub-scales comes from school-level averages.

## Supplementary Materials

The following are available online at https://www.mdpi.com/1660-4601/17/7/2194/s1, Modifications applied to MAPS Global data coding.
