# Supplementary material for: School Neighbourhood Built Environment Assessment for Adolescents’ Active Transport to School: Modification of an Environmental Audit Tool and Protocol (MAPS Global-SN)
Source: Int J Environ Res Public Health. 2020 Mar 25;17(7):2194. doi: 10.3390/ijerph17072194 (PMC7177319; doi:10.3390/ijerph17072194)
Supplement: Supplementary file 1 [file ijerph-17-02194-s001.pdf]

School Neighbourhood Built Environment Assessment for Adolescents' Active Transport to School: Modification of an Environmental Audit Tool and Protocol (MAPS Global-SN)

Adaptations to MAPS Global data coding

These changes refer to the MAPS Global Data Dictionary, Version September 6, 2017 and MAPS Global Audit Tool, Version 3/2/2016.

Route section, Aesthetics and Social sub-section:

- Item A4: *Are the buildings well-maintained? (%)*
- Item A5: *Is the landscape well maintained? (%)*
    - Adaptation: If no buildings or landscaping were present on the street segment side being audited, this item was left blank during coding.

Segment section

- Item S1_3: *Is a continuous sidewalk present?*
    - Adaptation: The recoding of this item (S1_3_recode, S1_3_negative) ultimately looks for the presence of a continuous sidewalk, therefore the modifications to these three items do not impact the overall item scoring, as the added response options were eventually recoded to 0 (in line with the item content description given). However, modifications were made to ensure consistency between the MAPS Global audit tool and the Data Dictionary coding.
      Modifications to the coding response options were made to reflect the initial response options given in the MAPS Global audit tool. Specifically, MAPS Global tool had four response options for the question, "Is a continuous sidewalk present?" (responses: 1. Yes, paved sidewalk is continuous; 2. No, paved sidewalk is not continuous; 3. No paved sidewalk, but informal walking path; 4. No, no sidewalk or informal walking path [skip to #10]). The Data Dictionary coding was modified from "No, no sidewalk=0", "Yes, sidewalk continuous=1", "No, not continuous=2" and revised to include the response options "No sidewalk/informal path (go to Q10)=0", "Yes, sidewalk continuous=1", "No, not continuous=2" and "Informal path=3".
- Item S1_3_recode: *Is a continuous sidewalk present? Recoded.*
    - Adaptation: The revised coding recoded the "No sidewalk/informal path (go to Q10)" and "Informal path" response options to "No sidewalk or informal path=0".
- Item S1_3_negative: *Is the sidewalk <u>continuous</u> within the segment? Recoded.*
    - Adaptation: The revised coding recoded the "No sidewalk or informal path" response option to "No=1".
- Item S1_15_16_0pts: *Either setback (S1_15, S1_16) >50 ft and no building.*
    - Adaptation: Item description modified to read, *Either setback (S1_15, S1_16) >50 ft **OR** no building*, as having a setback >50 ft **and** no building are mutually exclusive concepts.
- Item Overall_Segment: *Overall segment score*

    - Adaptation: The coding has been modified from “Segments_Pos_S1 – Sidewalk_Neg_S1” to reflect the actual item code of the Negative Segments Subscale, “Segments_Neg_S1”. The revised coding reads “Segments_Pos_S1 – Segments_Neg_S1”.

Crossing section

- Item C1_2: *Does this crossing take place on an overpass, underpass or bridge?*
    - Adaptation: MAPS Global audit tool includes a response option for “Not in working condition”. This response option was not originally included in the coding for item C1_2. This option has been added into the “No=0” response category.
- Item C1_3a: *Signalization: Pedestrian walk signals.*
    - Adaptation: MAPS Global audit tool includes a response option for “Pedestrian walk signal – signal not working”. This response option was not originally included in the coding for item C1_3a. This option has been added into the “No=0” response category.
- Item Curb_Qual_C1: *Curb quality and presence subscale*
    - Adaptation: Item code has been replaced with “CurbQual_C1” to reflect the code used in the “Positive Crossing Subscale (PosCrossChars_C1)”.
- Item CurbQual_C1: *Curb quality and presence subscale*
    - Adaptation: The C1_5 score has been added (+) to the other listed variables.

Cul-De-Sac section

Note: The cul-de-sac section score does not contribute to the MAPS Global overall scores and sub-scales.

- Item D1_1: *How close is the cul-de-sac or dead-end to participant’s home?*
    - Adaptation: Assessing the presence of a cul-de-sac in relation to a participant’s home was not possible when using MAPS Global to audit the school neighbourhood. A “Not Applicable” option was added to the response options and given the code of 0.
- Item D1_1_dichot: *How close is the cul-de-sac or dead-end to participant’s home? Dichotomized*
    - Adaptation: In the dichotomised item, the “Not Applicable” option remained coded as 0, along with the response options “<200 feet away” and “>200 feet away”.
- Item D1_3: *Can most of the cul-de-sac area be seen from participant’s home?*
    - Adaptation: A “Not Applicable” response option was created and coded as 0 (the same code as the “No” response option).

Cross-Domain Subscales

- Item Pedestrian_Infra: *Pedestrian Infrastructure subscale*
    - Adaptation: The item content description lists “low lights” as being included in the calculation of the Pedestrian Infrastructure subscale score; however, the item for “high streetlights” (S_MEAN_28_dichot) was listed in the scoring string. The revised scoring string includes the item “low streetlights” (S_MEAN_29_dichot).

- Item Pedestrian_Design: *Pedestrian Design subscale*
    - Adaptation: The item content description includes “kiosks,” but no kiosk code (SS5e) is included in the scoring string. Instead, the kiosk code appears in the *Bicycle Facilities* scoring string, but not in the related content description. The “kiosks” option was not relevant to *Bicycle Facilities*, so the revised scoring string included “kiosks” in the Pedestrian Design subscale.
